# Come

**Published:** 1890-09-13

**Authors:** 


### Words of Consolation.

### "COME."

Listen to the gracious invitation of the Blessed Jesus, "Come unto Me all ye that labour and are heavy laden." Why should we fear to come to Him? Is it because we believe Him to be a hard Master, who expects our debts to be paid to the uttermost farthing? How ignorant we are if such are our thoughts. Our dear Lord is the Good Shepherd, who knows His sheep by their names; none other is so gentle and so sweet as this Saviour, who longs after the souls of men. Yet they will not listen to Him, but scatter away from Him like a crowd of frightened sheep. Foolish hearts! why will you wander from One who loves you with an affection so deep and so true? Gather round His feet, for He is ever saying—

As He goes along His way,

O silly souls! come near Me;

My sheep should never fear Me;

I am the Shepherd true.

Perhaps you have heard this call when you were weary, wandering in the dreary ways of sin, the pleasures of which had ceased to satisfy. Did you put off till to-morrow to listen to His voice? Come now; come at once. It is God who invites you; His love is mighty! It is our Father; and His fondness for His children, the work of His own hand, goes far beyond anything we can dream of or imagine. True, He is a just God, but He is also a God of mercy, and His mercy is wider than the sea; it extends over all His works. Especially does He interest Himself in men who are made after His own likeness. He looks on their sorrows and failings with pity and compassion, and feels for them in the Person of His dear Son. He when on earth used His time and strength in doing good, and now that He is standing forever in heaven interceding for us, we know that He can be touched by a feeling for our infirmities, for He was tempted, as we are, *only without sin.*

This loving Saviour welcomes with joy the sinner who comes to Him in penitence and faith. The fountain of Christ's blood is always open to cleanse away his foulness and to heal the wounds and bruises he has received in wandering away from the fold. It was said of our Lord by the Prophet Isaiah that "He shall feed His flock like a shepherd; He shall gather the lambs with His arm, and carry them in His bosom." And He tells us of Himself, "I am the good shepherd, and I lay down my life for my sheep." Jesus stood and faced Satan, and fought for His sheep, and conquered the Evil One. Had He been only a hired servant He would have cared more for His own life than for the sheep, and would have run away when the wolf and the lion roared for their prey. But He is God, and all souls belong to Him by right. His banner over them is love, which will never be thrown down. If His sheep keep in His fold, and will answer to His voice, they will be safe; but, alas! they wander out into the bye-ways, and get into difficulties and disasters. Sometimes it is done in sheer wilfulness—they hate to be restrained—sometimes in ignorance, through following the multitude to do evil, just as one silly sheep will force its way through a gap in a hedge, and lead the rest of the flock after him.

The case is not hopeless even then, for "what man of you having an hundred sheep, if he lose one of them, will not leave the ninety and nine in the wilderness and go after that which is lost, until he find it? And when he hath found it, he layeth it on his shoulders rejoicing. And when he cometh home he calleth together his friends and neighbours, saying unto them rejoice with me, for I have found my sheep which was lost. Likewise joy shall be in heaven over one sinner that repenteth." Come, then, at once, so that when the Chief Shepherd shall appear ye shall be placed on His right hand among the flock, and receive a crown of glory that fadeth not away.

